# Low dose ketamine induces unique molecular and cellular responses in the brains of Alzheimer's disease mouse models

**DOI:** 10.1002/alz70855_100276

**Published:** 2025-12-23

**Authors:** Ricardo A S Lima‐Filho, Alinny Isaac, Cristovão De Lanna, Danielle Cozachenco, Gina C Couto, Ana Carolina Metello, Ícaro Raony, Sergio T. Ferreira, Fernanda Guarino De Felice, Mariana Boroni, Mychael V. Lourenco

**Affiliations:** ^1^ Federal University of Rio de Janeiro, Rio de Janeiro, Rio de Janeiro, Brazil; ^2^ Federal University of Rio de Janeiro, Rio de Janeiro, Brazil; ^3^ Brazilian National Cancer Institute (INCA), Rio de Janeiro, Brazil; ^4^ Oswaldo Cruz Foundation, Rio de Janeiro, Brazil; ^5^ Federal University of Rio de Janeiro, Rio de Janeiro, RJ, Brazil; ^6^ D'Or Institute for Research and Education, Rio de Janeiro, RJ, Brazil; ^7^ Queen's University, Kingston, ON, Canada; ^8^ Universidade Federal do Rio de Janeiro, Rio de Janeiro, RJ, Brazil

## Abstract

**Background:**

Ketamine is a non‐competitive *N*‐methyl‐d‐aspartate‐type glutamate receptor antagonist mainly used as an anesthetic. In addition to its synaptic effects, ketamine controls signaling pathways linked to mRNA translation and neuroinflammation. In low doses, ketamine has been employed as a rapidly acting long‐lasting antidepressant with considerable efficacy even in treatment resistant patients. Major depressive disorder (MDD) is an important risk factor and comorbidity of Alzheimer's disease (AD) and dementia. Synaptic dysfunction, loss of proteostasis and neuroinflammation are hallmarks of AD, but the impact of ketamine in the context of AD has been overlooked.

**Methods:**

We investigated how ketamine affects pathological markers of AD in two mouse models. A single dose of ketamine (10 mg/Kg) was administered to amyloid‐β oligomer (AβO) injected mice. Transgenic APP/PS1 mice were chronically treated with ketamine for 35 days. Memory function and hippocampal amyloid pathology and glial morphology were evaluated. Transcriptomic analysis (RNA sequencing) was also performed with wildtype (WT) and APP/PS1 mice hippocampi.

**Results:**

Low‐dose ketamine protected mice from the memory impairment induced by AβO injection. In transgenic APP/PS1 mice, chronic treatment had no impact on memory. Notably, transcriptomic analyses revealed that chronic ketamine induced a transcriptional profile in wildtype (WT) mice with significant similarity to the APP/PS1 genotype. Enrichment analysis of the convergent >700 differentially expressed genes (DEGs) in these groups uncovered pathways related to synaptic transmission and protein translation. Moreover, APP/PS1 and WT mice chronically treated with ketamine had >200 DEGs in common but modulated in opposite directions – while only 12 DEGs regulated in convergent directions. Enrichment analysis revealed that the most significant pathways among oppositely regulated DEGs are related to glial function. Immunohistological analyses showed that ketamine increased microglial corralling around amyloid plaques and promoted opposite changes in microglial morphology in APP/PS1 and WT hippocampi.

**Conclusions:**

Our data show that chronic low dose ketamine induces a unique transcriptional and cellular response in the context of AD. Moreover, ketamine induces AD‐like transcriptional signature in WT mice. These results indicate that ketamine may modulate AD risk and underscore the need for caution when considering low‐dose ketamine in patients at risk for or diagnosed with AD.

